# Adsorption Performance for Reactive Blue 221 Dye of β-Chitosan/Polyamine Functionalized Graphene Oxide Hybrid Adsorbent with High Acid–Alkali Resistance Stability in Different Acid–Alkaline Environments

**DOI:** 10.3390/nano10040748

**Published:** 2020-04-14

**Authors:** Chih-Wei Chiu, Ming-Tsung Wu, Chih-Lung Lin, Jia-Wun Li, Chen-Yang Huang, Yu-Chian Soong, Jimmy Chi-Min Lee, William Anderson Lee Sanchez, Hsuan-Yu Lin

**Affiliations:** Department of Materials Science and Engineering, National Taiwan University of Science and Technology, Taipei 10607, Taiwan; d10404011@mail.ntust.edu.tw (M.-T.W.); jerry@email.ctci.org.tw (C.-L.L.); a12352335@yahoo.com.tw (J.-W.L.); d10504015@gapps.ntust.edu.tw (C.-Y.H.); yuchiansoong@gmail.com (Y.-C.S.); jimmy@cleaninst.com (J.C.-M.L.); williaxom@gmail.com (W.A.L.S.); dashingcodyrhodes728@gmail.com (H.-Y.L.)

**Keywords:** graphene oxide, β-chitosan, adsorption, reactive blue 221 dye, effluent treatment

## Abstract

A hybrid material obtained by blending β-chitosan (CS) with triethylenetetramine-functionalized graphene oxide (TFGO) (CSGO), was used as an adsorbent for a reactive dye (C.I. Reactive Blue 221 Dye, RB221), and the adsorption and removal performances of unmodified CS and mix-modified CSGO were investigated and compared systematically at different pH values (2, 3, 4, 5, 6, 7, 8, 9, 10, 11, and 12). The adsorption capacities of CS and CSGO were 45.5 and 56.1 mg/g, respectively, at a pH of 2 and 5.4 and 37.2 mg/g, respectively, at a pH of 12. This indicates that TFGO was successfully introduced into CSGO, enabling π–π interactions and electrostatic attraction with the dye molecules. Additionally, benzene ring-shaped GO exhibited a high surface chemical stability, which was conducive to maintaining the stability of the acid and alkali resistance of the CSGO adsorbent. The RB221 adsorption performance of CS and CSGO at acidic condition (pH 3) and alkaline condition (pH 12) and different temperatures was investigated by calculating the adsorption kinetics and isotherms of adsorbents. Overall, the adsorption efficiency of CSGO was superior to that of CS; thus, CSGO is promising for the treatment of dye effluents in a wide pH range.

## 1. Introduction

Water is a critical resource for life, and inadequate drinking water and sanitation are becoming problems for humans. Additionally, the global hydrosphere system—including lakes, rivers, and oceans—is suffering from the effluents produced by industrial activities, and the discharge of urban domestic effluents into the natural hydrosphere system has increased the concentrations of hazardous pollutants. Among the many types of effluents, dye effluents should attract particular attention owing to their characteristics of high chroma, high toxicity, and difficulty of degradation, which can result in severe environmental pollution and damage. Most organic dyes have cumulative effects on organisms [1], can induce carcinogenesis [2,3,4], and may severely harm the human body, e.g., causing abnormal kidney functions and damaging the reproductive system, liver, brain, and central nervous system [5,6]. The primary pollution sources of dye effluents include textile dyeing and finishing [7,8,9], printing and dyeing [10], and leather processing industries [11]. Each year, more than 10,000 commercial synthetic dyes are used in dyeing processes. Among the various dyes—which have a total global market output of more than 7 × 10^5^ metric tons—reactive dyes account for approximately 50% of the total annual use. Approximately 5–10% of the dyes form industrial effluents in the dyeing process [12,13]. In general, a reactive dye (C.I. Reactive Blue 221 Dye, RB221) widely used in textile industry. The RB221 could be produce harmful by products after any simple decolouring process [14]. Therefore, the effective removal of these dyes from effluents is highly important.

Various technologies have been developed to remove dyes from dye effluents for mitigating their environmental hazards. Common methods include the adsorption method [15,16], ion exchange [17], membrane filtration [18,19], the electrochemical method [20], the Fenton method [21,22], the advanced oxidation method [23,24], and the biological treatment method [25]. The adsorption method employs the principle of adsorption to remove dye contaminants; it is easy to implement and has a high removal efficiency [26]. In recent years, many studies have been performed on the use of different adsorption materials to remove dyes from effluents. For example, graphene oxide (GO) has been verified; it has a large specific surface area, and there are various oxidizing functional groups (e.g., hydroxyl, carboxyl, and epoxy) in its structure. Additionally, it exhibits good dispersibility and an excellent adsorption force in water [27,28]. The most significant technical challenge of applying GO for adsorption is the difficulty of solid–liquid separation after adsorption; the separation is performed with ultrahigh-speed centrifuges, but it takes a long time to collect the GO, which is highly dispersed in water [29]. To resolve the aforementioned shortcomings, the GO adsorption mainly focuses on introducing magnetic separation technology to GO, that is, solving the solid–liquid separation problem through the synthesis of hybrid materials containing magnetic nanoparticles [30]. In particular a molecular scale dispersion, a suitable stabilizing agent is required so as to maintain a homogenous suspension [31]. Additionally, nanocomposites can be synthesized by mixing polymers and GO to improve the adsorption efficiency of GO [32,33].

Chitosan is obtained via the chemical treatment of chitin. The amine and hydroxyl groups in the molecular structure of chitosan can serve as the active chelation sites for adsorbing dye molecules [34]. Notably, the adsorption of dye molecules can involve different mechanisms (the intermolecular force may be an electrostatic attraction, a π–π interaction, or H bonding) and depends on the solution composition, pH value, and chemical structure and morphology of the dye. Studies have revealed that chitosan has a good affinity to most dyes. Chitin, chitosan, and chitosan derivatives have been investigated for dye removal [35,36]. Chitosan mainly relies on electrostatic attraction to desorb and remove acidic anionic dyes, because the amine functional groups in its molecular structure can be protonated into cationic groups under the adsorption conditions of an acidic aqueous solution. Thus, chitosan has a high adsorption capacity for acidic anionic dyes [37]; however, the chitosan adsorbent is decomposed in acidic aqueous solutions [38]. To synthesize chitosan that is resistant to acid hydrolysis and to improve its ability to remove acidic anionic dyes, chemical grafting modification can be employed to enhance the acid-resistance stability of chitosan in low-pH solutions. Our recent research suggests that the adsorption capacity of β-chitosan (CS) can be increased from 45.571 mg/g (unmodified CS) to 52.417 mg/g (modified CS, i.e., CS crosslinked with triethylenetetramine, BCCT). This is because the amine functional groups in the modified CS molecular structure can be protonated to cationic groups when exposed to a large amount of H^+^, improving the removal and adsorption of acidic anionic dyes. However, the adsorption capacity of BCCT after modification was only 10 mg/g in an alkaline adsorption environment (pH = 12). This is because a large amount of OH^−^ in water reduces the protonation degree of the amine functional groups of BCCT, significantly reducing the removal and adsorption of acidic anionic dyes [39]. It is imperative to overcome these disadvantages.

To address the foregoing issues, we blended CS and triethylenetetramine-functionalized GO (TFGO) into a hybrid material to improve the adsorption capacity of chitosan under alkaline conditions and solve the solid–liquid separation problem after GO adsorption. The adsorption effects of adsorbents for Reactive Blue 221 dye (RB221) before and after mixing CS with TFGO were compared under different pH values, adsorption temperatures, adsorption durations, and initial dye concentrations. Additionally, the adsorption kinetics and isotherms of adsorbents were analyzed to understand the mechanisms of adsorption behavior.

## 2. Materials and Methods

### 2.1. Materials

**Figure 1:**
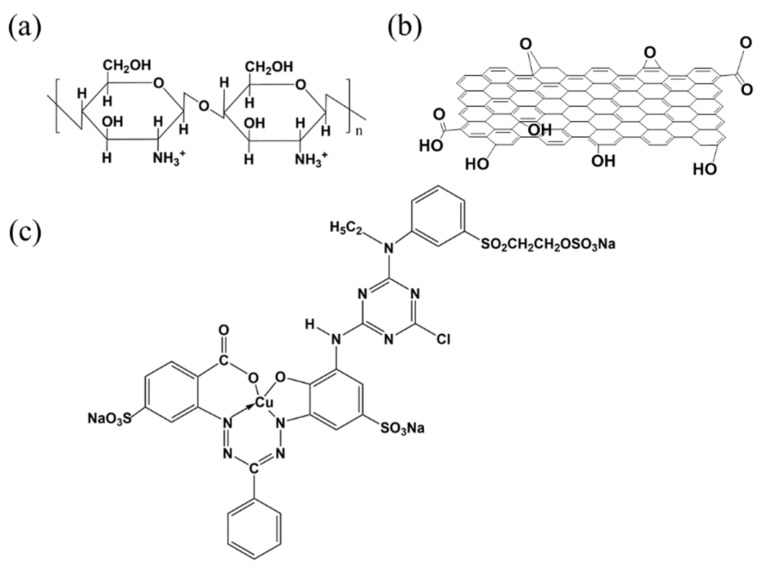
Chemical structures of (**a**) β-chitosan (CS), (**b**) graphene oxide (GO), and (**c**) reactive Blue 221 Dye (RB221).

Food-grade CS with a deacetylation degree of 90% and a molecular weight of 500 KD was purchased from Charming and Beauty Co., Taipei, Taiwan. Technical-grade graphene oxide (GO) with an O content of 45% was purchased from E Way Technology, Kaohsiung, Taiwan. Reagent-grade triethylene tetramine (TETA, 60%) was purchased from Acros, Morris Plains, NJ, USA. Reagent-grade hydrochloric acid (HCl, 37%) was purchased from SCHARLAU, Barcelona, Spain. Reagent-grade sodium hydroxide (NaOH, 98.5%) was purchased from Acros, Morris Plains, NJ, USA. Reagent-grade dimethyl sulfoxide (DMSO) was purchased from Merck, Darmstadt, Germany. Technical-grade CI Reactive Blue 221 dye (RB221) with a molecular weight of 890 g/mol was purchased from Nippon Kayaku Co., Ltd., Tokyo, Japan. The chemical structures of CS, GO, and RB221 are shown in Figure 1.

### 2.2. Synthesis of TFGO and CS/TFGO Hybrid Adsorbent (CSGO)

Triethylenetetramine-functionalized graphene oxide (TFGO) was synthesized via a previously reported procedure [40]. First, 20 mg of GO was dispersed in 20 mL of deionized (DI) water under ultrasonic vibration for 1 h; then, 0.2 mL of TETA and 0.2 mL of 1N lye were added to react at 60 °C for 6 h. Subsequently, the obtained product was separated via centrifugation and washed repeatedly with DI water to neutrality, yielding the end product: TFGO. The synthesized TFGO product, 1 g of CS, and 100 mL of DI water were added to a single-neck reaction flask. The obtained suspension was evenly dispersed via ultrasonic vibration and then stirred and dispersed at room temperature for 8 h. Then, the suspension was filtered via suction, and the filter cake was dried in an oven at a constant temperature of 80 °C to obtain the CS/TFGO hybrid adsorbent (CSGO). Finally, the structures of GO and TFGO were verified using Fourier transform infrared spectroscopy (FTIR), Raman spectroscopy, elemental analysis (EA), and transmission electron microscopy (TEM) (Supplementary Material Figures S1–S3 and Tables S1–S3).

### 2.3. Test of Reactive Dye Adsorption

The adsorption effects of adsorbents for RB221 were tested under different conditions, such as different pH values, adsorption temperatures, adsorption durations, and initial dye concentrations.

The effect of the pH (range of 2–12) on the adsorption capacity of the adsorbent was investigated at room temperature. First, 25 mL of an RB221 solution (concentration of 600 mg/L) and 0.25 g of the adsorbent were added to a reaction flask. Then, the suspension was magnetically stirred at 800 rpm for 30 min and centrifuged for 15 min at 6000 rpm to separate the adsorbent from the aqueous solution; the supernatant was extracted to analyze the final residual concentration.

The isothermal adsorption mode of the adsorbent was tested at different adsorption temperatures: 303, 313, and 323 K. First, 0.25 g of the adsorbent and 25 mL of RB221 solution—with initial concentrations of 3000, 3400, 3800, 4200, 4600, and 5000 mg/L at a pH of 3.0, as well as concentrations of 1000, 1400, 1800, 2200, 2600, and 3000 mg/L at a pH of 12.0, respectively—were added to a reaction flask. Then, the suspensions were magnetically stirred at 800 rpm for 6 h and centrifuged for 15 min at 6000 rpm to separate the adsorbents from the aqueous solutions. The supernatants were extracted to measure the final residual concentration.

Next, the adsorption kinetics of the adsorbent were tested at different adsorption temperatures: 303 and 333 K. First, 0.25 g of the adsorbent and 25 mL of RB221 solution—with a concentration of 2000 mg/L at pH 3.0 and a concentration of 1000 mg/L at pH 12.0, respectively—were added to a reaction flask. Then, the suspensions were magnetically stirred at 800 rpm for 15, 30, 45, 60, 90, 120, 150, and 180 min. Subsequently, the suspensions were centrifuged for 15 min at 6000 rpm to separate the adsorbent from the aqueous solutions, and the supernatants were extracted for concentration analysis.

The concentrations of the extracted supernatants were analyzed using ultraviolet–visible (UV–vis) spectroscopy (V-630 mode Spectrophotometer, JASCO Corporation, Tokyo, Japan). The equilibrium adsorption capacity of the adsorbent for RB221 was calculated using Equations (1) and (2) [41]:

(1) $$q_{e}=\frac{\left(C_{0}-C_{e}\right)\cdot V}{m}$$

(2) $$q_{t}=\frac{\left(C_{0}-C_{t}\right)\cdot V}{m}$$

Here, *q_e_* represents the amount of adsorbed RB221 at the adsorption equilibrium (mg/g), *q_t_* represents the amount of adsorbed RB221 at adsorption time *t* (mg/g), *C_0_* represents the initial concentration of RB221 in the liquid phase (mg/L), *C_e_* represents the concentration of RB221 in the liquid phase at equilibrium (mg/L), C*_t_* represents the concentration of RB221 at adsorption time *t* (mg/L), *V* represents the volume of the RB221 solution (L), and *m* represents the mass of the adsorbent used (g).

### 2.4. Characterization and Measurements

#### 2.4.1. Fourier Transform Infrared Spectroscopy (FTIR)

First, the powder sample was dried and dehydrated in a 100 °C oven for 24 h. The dried sample was then ground in an agate mortar. Next, potassium bromide (KBr, spectroscopic grade) power was added, and the powders were ground further until mixed evenly. The mixed powders were then pressed into pellets for FTIR analysis. The number of scans was set as 32 for each sample during FTIR spectroscopy.

#### 2.4.2. Ultraviolet–Visible (UV–Vis) Spectroscopy 

First, the powder sample was dried and dehydrated in a 100 °C oven for 24 h. Then, a certain amount of the sample was placed into the fixture. For a liquid sample, a sample vial was used to hold a certain amount of the liquid. During the collection of the ultraviolet (UV) spectra for both sample types, the number of scans was set as 32 for each sample.

#### 2.4.3. Elemental Analysis (EA) 

The to-be-tested sample was first dried and dehydrated in a 100 °C oven for 24 h. Then, approximately 3 to 5 mg of the dried sample was placed in a tin capsule and folded into a small cube, which was placed in an automated sampler for analysis.

#### 2.4.4. Transmission Electron Microscopy (TEM) 

The sample was added to DI water and dispersed under ultrasonic vibration for 30 min. The dispersion was then dripped onto a Cu mesh, which was dried and placed under a TEM for observation.

#### 2.4.5. Raman Spectroscopy 

The sample was added to DI water and dispersed under ultrasonic vibration for 30 min. The dispersion was then dripped onto an Al plate and placed on a hotplate to dry. After drying, it was placed in a Raman spectrometer for observation. The selected laser beam had a wavelength of 532 nm, and the scanning wavelengths ranged from 400 to 4000 cm^−1^ at room temperature.

#### 2.4.6. Field-Emission Scanning Electron Microscopy (FE-SEM)

The sample was added to DI water and dispersed under ultrasonic vibration for 30 min. The dispersion was dripped onto a glass substrate and dried on a hotplate. After drying, it was mounted onto the SEM stage with carbon tape and placed in the SEM vacuum chamber for observation.

## 3. Results and Discussion

### 3.1. Adsorption Capacities of Adsorbents for RB221 Affected by Changing pH Values

**Figure 2:**
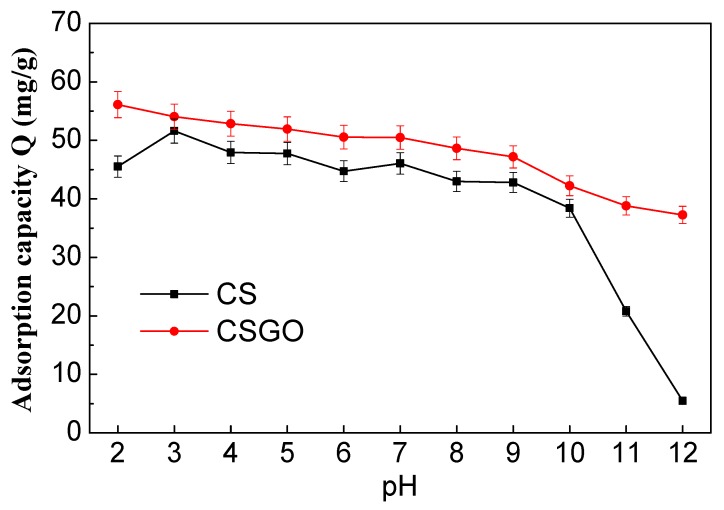
Adsorption capacities of the β-chitosan (CS) and β-chitosan/triethylenetetramine-functionalized graphene oxide (CSGO) adsorbents for C.I. Reactive Blue 221 Dye (RB221) with respect to the pH.

**Figure 3:**
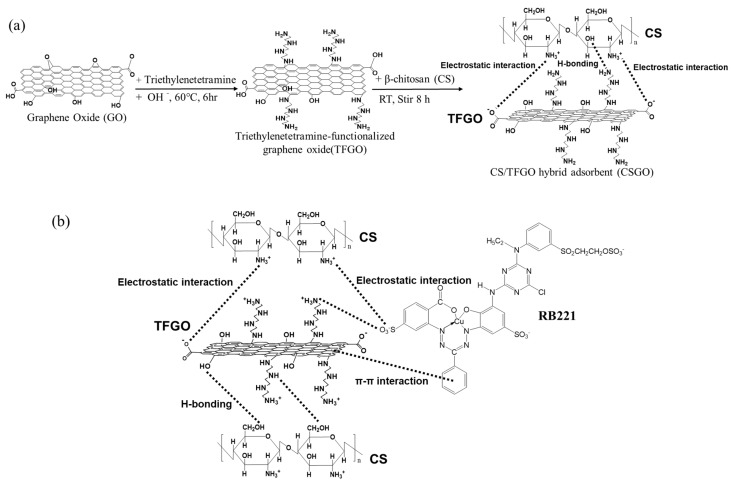
(**a**) Synthesis of triethylenetetramine-functionalized graphene oxide (TFGO) and the intermolecular forces between CS and TFGO during blending. (**b**) Mechanism of intermolecular forces during the adsorption of an organic dye (RB211) by the CS/TFGO hybrid adsorbent.

RB221 containing sulfonate groups was used as the dye in this study. An RB221 solution with an initial pH of approximately 5.5 was synthesized initially, which was then adjusted with 2 N, 1 N, 0.5 N, and 0.1 N NaOH or HCl solutions to obtain the required pH conditions (pH 2 to 12). The adsorption results of RB221 for CSGO at the designed weight ratios of CS/TFGO, 0:1, 5:1, 50:1, and 1:0 are presented in Figure S4 (Supplementary Materials). The adsorption of RB221 by the various CSGO adsorbents was influenced to a great capacity by the pH. The amounts of dye adsorbed per gram of each adsorbent, CS/TFGO with weight ratios of 0:1, 5:1, 50:1, and 1:0, at the strongly acidic condition of pH 2 were 4.8, 50.2, 56.1, and 45.5 mg/g, respectively. The adsorption capacity of the adsorbents for the dye dropped sharply in the alkaline solutions of pH 7 to 12. The CS/TFGO with weight ratio of 50:1 showed the best adsorption for the dyes at all pH. Owing to the benzene ring-shape, GO exhibited a high acid–alkali resistance, which was promoted to maintaining the stability of the acid and alkali resistance of the CSGO adsorbent. Furthermore, Figure 2 shows detailedly the effects of the pH on the adsorption capacities of different adsorbents for RB221. The pH condition had a significant effect on the removal effects of the adsorbents CS and CSGO for RB221. Under an extremely acidic condition (pH 3), the maximum dye removal capacities were measured to be 51.5 and 54.0 mg/g, respectively. As the pH increased from 3 to 9, the dye adsorption capacities of the adsorbents slowly decreased; when the pH further increased from 10 to 12, a sudden decrease in the adsorption capacity was observed for CS. This adsorption behavior of CS for organic dyes mainly resulted from the Coulomb electrostatic interaction between the dye’s anionic sulfonate group (SO_3_^−^) and the chitosan’s cationic protonated amine group (–NH_3_^+^), as indicated by Equation (3) [42]. The sodium sulfonate groups (R–SO_3_Na) on RB221 were dissociated into activated anionic sulfonate groups (R–SO_3_) in water, as indicated by Equation (4). Additionally, in a low-pH solution, more amine groups on the adsorbents were protonated, as indicated by Equation (5) [43], increasing the number of dye molecules adsorbed by the adsorbents. However, an excessive increase in the acidity of the dye solution (pH 2) reduces the adsorption effect for the dye. This is because chitosan is over-protonated by H ions (H^+^) in an extremely low-pH environment, leading to acidic degradation. The adsorption mechanisms of the CS/TFGO hybrid adsorbent for organic dye RB221, which include π–π stacking interaction, H bonding, and electrostatic force, are shown in Figure 3 [44,45]. After hybrid blending, the obtained CSGO adsorbent can leverage the π–π interaction, H bonding, electrostatic, and other forces of TFGO to achieve a good adsorption effect at a low pH. In a highly acidic environment with a pH of 3, the removal effects/adsorption capacities of the adsorbents CS and CSGO for RB221 were measured to be 51.6 and 54.04 mg/g, respectively. Therefore, the adsorption efficiency of the modified CS (CSGO) was stable and exhibited the best performance, and the adsorption efficiency of unmodified CS declined remarkably. In a highly alkaline environment with a pH of 12, the removal effects/adsorption capacities of the adsorbents CS and CSGO for RB221 were measured to be 5.4 and 37.2 mg/g, respectively. CSGO exhibited a higher capture efficiency for dye molecules than CS because the cationic protonated amine groups (–NH_3_^+^) of the CS adsorbent were affected by strong base ions and converted into amine groups (–NH_2_), reducing its ability to capture dye molecules, whereas in the case of the CSGO adsorbent, the π–π interaction, H bonding, electrostatic force, and other forces of the TFGO facilitated the capture of the dye molecules.
(3)
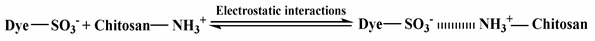

(4) $$\mathrm{Dye}―SO_{3}\mathrm{Na}\overset{H_{2}O}{\underset{\mathrm{Dissociation}}{⇌}}\mathrm{Dye}―{\mathrm{SO}}_{3}{}^{-}+H^{+}$$

(5) $$\mathrm{Chitosan}―NH_{2}\overset{H^{+}}{\underset{\mathrm{Protonation}}{⇌}}\mathrm{Chitosan}―NH_{3}{}^{+}$$

### 3.2. Adsorption Mechanism of CSGO for RB221

#### 3.2.1. H Bonding Forces

**Figure 4:**
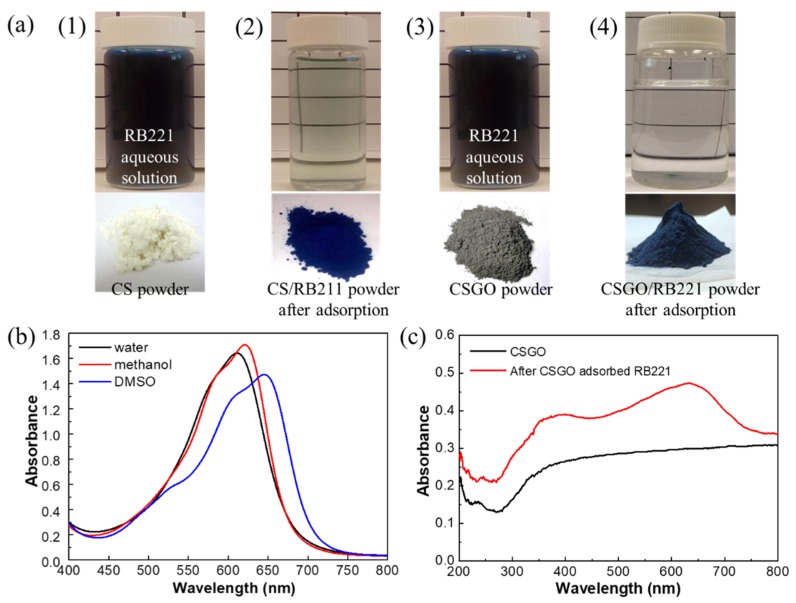
(**a**) Photographs of RB221 aqueous solutions and adsorbents before and after RB221 was adsorbed by different adsorbents: (1) RB221 solution and CS powder before adsorption, (2) RB221 solution and CS/RB211 powder after adsorption; (3) RB221 solution and CSGO powder before adsorption, (4) RB221 solution and CSGO/RB221 powder after adsorption. UV–vis electron absorption spectra: (**b**) changes in UV–vis spectra of RB221 solutions (concentration of 1.14 × 10^−3^ mol/L) prepared using different solvents (such as water, methanol, and DMSO); (**c**) comparison of CSGO spectra obtained before and after adsorbing RB221.

Figure 4a (1 and 2) and Figure 4a (3 and 4) show photographs of RB221 solutions as well as the adsorbent dry powders and adsorbent/RB221 precipitations before and after RB221 was adsorbed by CS and CSGO, respectively. In addition, Supplementary Material Video S1 show the RB221 adsorption of adsorbents for dye ion before and after adsorption. Before treatment with the adsorbents, the aqueous dye solution appeared dark blue. After the dye was adsorbed by CS, the aqueous solution appeared to be transparent pale blue. Figure 4a (4) shows that after the adsorption of the dye with CSGO, the aqueous solution became almost transparent, indicating that CSGO had a better adsorption effect than CS. Additionally, the CS and CSGO adsorbent powders were pale yellow and dark gray, respectively, before the adsorption, but both turned into dark blue powders after the adsorption. Furthermore, FE-SEM was performed to examine the microstructure of the CSGO powder after the adsorption of RB221 (i.e., CSGO/RB221), and RB221 dye particles were observed on the CSGO surface (Supplementary Material Figure S5). 

To evaluate the H bonding strength between the dye and the adsorbent, the interactions between several polar solvents (such as water, methanol, and DMSO) and the dye in the experiment were analyzed. As shown in Figure 4b, DMSO (UV–Vis λ_max_ = 649.8 nm) is a non-H-bonding polar solvent. Because the electron energy level π* has a higher polarity than π, a polar solvent reduces the energy level of the π→π* transition, leading to a redshift in the signal compared with those of methanol (λ_max_ = 620.8 nm) and water (λ_max_ = 640 nm). Water is a dispersion medium with strong H bonding; thus, the UV–vis signal blue-shifted as RB221 dissolved. Figure 4c shows the changes in the UV–Vis spectra after the adsorption of RB221 by CSGO. After the CSGO adsorbed the RB221 dye, the UV–Vis signal shifted slightly toward a longer wavelength. This is because the RB221 molecule had a conjugate structure, absorbing energy in the UV region. As the number of conjugate bonds in the molecular structure increased, the absorption wavelength increased. Additionally, a hyperchromic effect was observed in CSGO after it adsorbed RB221, indicating that its structure changed significantly, which is consistent with the results of the previously reported isothermal adsorption model and adsorption kinetics model [46].

#### 3.2.2. FTIR Analysis

**Figure 5:**
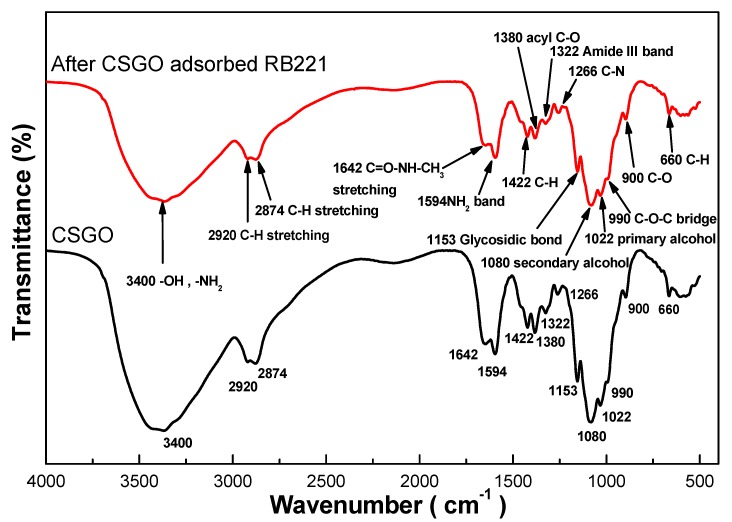
Changes in the IR spectra of CSGO after the adsorption of RB221.

Figure 5 shows the changes in the IR spectra of CSGO before and after the adsorption of RB221. After the adsorption of RB221, the signal strengths at the characteristic peaks for the primary alcohol and secondary alcohol functional groups on the modified CSGO molecules were significantly reduced. The decreasing in signal strengths at the wavenumbers of primary alcohol (1022 cm^−1^), secondary alcohol (1080 cm^−1^), and glycosidic (1153 cm^−1^) functional groups indicate that adsorption bonds were formed at the corresponding positions. Additionally, the signals strength at the wavenumbers corresponding to the amide (2874 and 2920 cm^−1^) and amine (1642 and 3400 cm^−1^) functional groups were observed, indicating that a large amount of dye amine groups was adsorbed on CSGO, which confirmed the adsorption of RB221 by CSGO. Additional qualitative results for CSGO before and after the adsorption (obtained using infrared (IR) spectra) are presented in Table S4 (Supplementary Material). Furthermore, the fine distribution of the TFGO in the CS matrix was evidenced by the FE-SEM observation. The homogeneous distribution of TFGO platelet in the CS matrix is demonstrated (Supplementary Material Figure S6).

### 3.3. Discussion of Isothermal Adsorption Models

The three most commonly used isotherms for describing isothermal adsorption are the Langmuir model, Freundlich model, and Temkin model. In this study, the isothermal adsorption behaviors of different adsorbent materials were analyzed.

#### 3.3.1. Langmuir Adsorption Isotherm

The Langmuir adsorption isotherm assumes a homogeneous surface covered by a monolayer, and there is no interaction between the adsorbate and its adjacent substances. The nonlinearity and linearity of the model can be expressed by Equations (6) and (7):

(6) $$q_{e}=\frac{Q_{0}bC_{e}}{1+bC_{e}}$$

(7) $$\frac{1}{q_{e}}=\frac{1}{Q_{0}}+\frac{1}{Q_{0}bC_{e}}$$

where *q_e_* represents the amount of RB221 adsorbed per gram of adsorbent at equilibrium (mg/g), *Q_0_* represents the monolayer adsorption capacity (mg/g), *C_e_* represents the equilibrium concentration of RB221 (mg/L), and *b* represents the Langmuir adsorption isotherm constant (L/mg).

**Figure 6:**
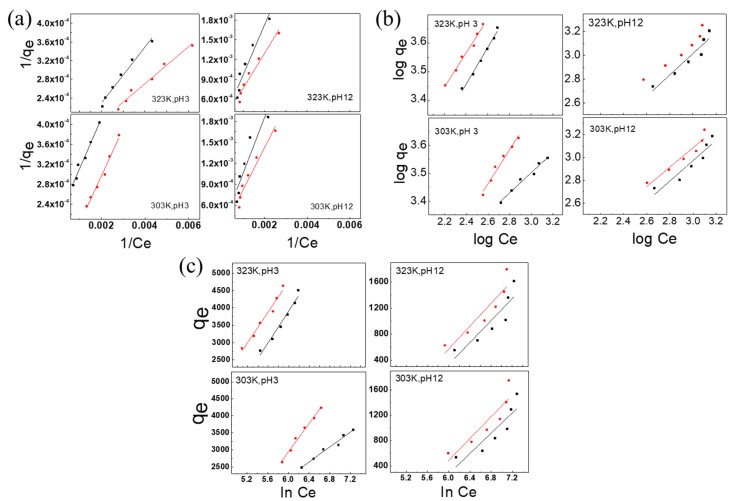
Isothermal adsorption models for the two adsorbent materials (■ CS, ● CSGO) at different temperatures (303 and 323 K): (**a**) Langmuir isotherm; (**b**) Freundlich isotherm; (**c**) Temkin isotherm.

The relationship between 1/*q_e_* and 1/*C_e_* was plotted, as shown in Figure 6a. The values of *b* and *Q*_0_ were given by the slope and y-intercept, respectively. *Q*_0_ represents the monolayer adsorption capacity for the dye (mg/g), and *b* is the Langmuir adsorption isotherm constant (L/mg). The basic characteristic of the Langmuir isotherm can be represented by the equilibrium constant *R_L_*, which is a dimensionless constant referred to as the separation factor that is used for predicting the nature of the adsorption process, and is expressed in Equation (8).

(8) $$R_{L}=\frac{1}{1+bC_{0}}$$

Here, *R_L_* represents the Langmuir separation factor (L/mg), *b* represents the Langmuir adsorption isotherm constant (L/mg), *C*_0_ represents the initial concentration of RB221 (mg/L), and *R_L_* indicates the nature of the adsorption process: *R_L_* > 1 corresponds to an unfavorable adsorption condition, *RL* = 1 corresponds to linear adsorption, 0 < *R_L_* < 1 corresponds to favorable adsorption conditions, and *R_L_* = 0 corresponds to irreversible adsorption.

Using the data in Table 1, the adsorption models of the adsorbents before and after blending modification at different pH values and temperatures were obtained. The results indicated that when the temperature increased from 303 to 323 K, at a pH of 3, the monolayer adsorption capacities (*Q*_0_) of CS and CSGO for RB221 increased from 9.94 and 16.61 mg/g to 10.53 and 25.32 mg/g, respectively. At a pH of 12, the monolayer adsorption capacities (*Q*_0_) of CS and CSGO for RB221 increased from 1.27 and 1.68 mg/g to 1.32 and 1.89 mg/g, respectively. Thus, at 303 K, CSGO had a higher monolayer adsorption capacity than CS; even when the temperature increased to 323 K, its adsorption capacity was higher.

Analyzing the separation factor *R_L_* for the adsorption of RB221 by adsorbents at different pH values and temperatures revealed that when the temperature increased from 303 to 323 K, at a pH of 3, the *R_L_* values of CS and CSGO changed from 6.6 × 10^−7^ and 3.5 × 10^−7^ to 5.5 × 10^−7^ and 8.4 × 10^−7^, respectively. At a pH of 12, the *R_L_* values of CS and CSGO changed from 3.8 × 10^−7^ and 2.6 × 10^−7^ to 3.36 × 10^−7^ and 3.77 × 10^−7^, respectively. Thus, both CS and CSGO promoted the adsorption process. It is inferred that CSGO mainly utilized the graphene π–π interaction to capture RB221 in the alkaline environment; thus, the separation factor *R_L_* for the CSGO adsorption process increased with the temperature.

#### 3.3.2. Freundlich Adsorption Isotherm

The Freundlich adsorption isotherm is used to describe the adsorption characteristics of heterogeneous surfaces and is expressed by Equations (9) and (10).

(9) $$Q_{e}=K_{f}C_{e}{}^{\frac{1}{n}}$$

(10) $$\log Q_{e}=\log K_{f}+\frac{1}{n}\log C_{e}$$

Here, *Q_e_* represents the amount of RB221 adsorbed per gram of adsorbent at equilibrium (mg/g), *K_f_* represents the Freundlich adsorption isotherm constant (mg/g), *n* represents the Freundlich adsorption strength, and *C_e_* represents the equilibrium concentration of RB221 (mg/L).

The relationship between log*Q_e_* and log*C_e_* was plotted, as shown in Figure 6b. The values of 1/*n* and *K_f_* were given by the slope and y-intercept, respectively. *K_f_* represents the approximate adsorption capacity. The value of 1/*n* indicates whether the adsorption process is favorable: when *n* > 1, the adsorption is favorable.

According to the data in Table 1, the approximate adsorption capacities (*K_f_*) of the adsorbents for Rb221 before and after blending modification at different pH values and temperatures were determined. When the temperature increased from 303 to 323 K, at a pH of 3, the *K_f_* values of CS and CSGO changed from 259.24 and 74.11 mg/g to 82.47 and 132.13 mg/g, respectively. At a pH of 12, the *K_f_* values of CS and CSGO changed from 1.99 and 3.49 mg/g to 2.13 and 4.37 mg/g, respectively.

At 303 K, the approximate adsorption capacity of CSGO at a pH of 3 was slightly insufficient; however, when the temperature increased to 323 K, it exhibited better performance than CS. When the temperature increased from 303 to 323 K, at a pH of 3, the adsorption strengths *n* of CS and CSGO changed from 2.75 and 1.63 mg/g to 1.55 and 1.66 mg/g, respectively. At a pH of 12, the *n* values of CS and CSGO changed from 1.12 and 1.17 mg/g to 1.11 and 1.20 mg/g, respectively. At 303 K, the *n* value for the adsorption capacity of CSGO at a pH of 3 was slightly insufficient; however, when the temperature increased to 323 K, CSGO exhibited better performance than CS. 

#### 3.3.3. Temkin Adsorption Isotherm

The Temkin adsorption isotherm model assumes that the adsorption energy is related to the coverage and is expressed as follows:

(11) $$q_{e}=B\ln K_{t}+B\ln C_{e\prime }$$

where *q_e_* represents the amount of RB221 adsorbed per gram of adsorbent at equilibrium (mg/g), *K_t_* represents the equilibrium binding constant corresponding to the maximum binding energy (dm^3^/g), *b* is a constant related to the heat of adsorption (J/mol), and *C_e_* represents the equilibrium concentration of RB221 (mg/L).

The relationship between *q_e_* and ln*C_e_* was plotted, as shown in Figure 6c, and the values of *b* and *K_t_* were given by the slope and y-intercept, respectively.

According to the data in Table 1, the values of the equilibrium binding constant *K_t_* corresponding to the maximum binding energy for the adsorption of RB221 by adsorbents before and after blending modification at different pH values and temperatures were determined. When the temperature increased from 303 to 323 K, at a pH of 3, the *K_t_* values of CS and CSGO changed from 1.28 and 1.24 dm^3^/g to 1.26 and 1. 29 dm^3^/g, respectively. At a pH of 12, the *K_t_* values of CS and CSGO changed from 1.19 and 1.19 dm^3^/g to 1.20 and 1.20 dm^3^/g, respectively. 

Regarding the heat of adsorption *b* for the process of adsorption of RB221 by the adsorbents, when the temperature increased from 303 to 323 K, at a pH of 3, the *b* values of CS and CSGO changed from 0.58 and 0.26 J/mol to 0.27 and 0.32 J/mol, respectively. At a pH of 12, the *b* values of CS and CSGO changed from 0.55 and 0.53 J/mol to 0.56 and 0.57 J/mol, respectively. Thus, it can be inferred that in an acidic environment, the adsorption of CS was significantly affected by the temperature. In contrast, for CSGO, in an alkaline environment, little difference in the overall changes of the adsorption heat was observed between the two adsorbents. It could be that the cationic protonated amine groups (–NH^3+^) on the two adsorbent molecules turned into amine groups (–NH_2_) under the influence of the strong base ions, thereby losing the capturing effects for dye molecules. 

### 3.4. Adsorption Thermodynamics

Thermodynamic experiments were conducted to study the adsorption of RB221 by different adsorbents at a pH of 12 and an initial RB221 concentration of 1800 mg/L and a pH of 3 and an initial RB221 concentration of 3800 mg/L. The thermodynamic parameters related to the adsorption process—such as the standard free energy change (Δ*G*^0^), standard enthalpy change (Δ*H^0^*), and standard entropy change (Δ*S*^0^)—were calculated using the van’t Hoff equations (Equations (12) and (13)) [41].

(12) $$\Delta G^{0}=-RT\ln K_{c}$$

(13) $$\ln K_{c}=-\frac{\Delta H^{0}}{RT}+\frac{\Delta S^{0}}{R}$$

where *K_C_* represents the distribution coefficient at different temperatures, which is given by (*q_e_*/*C_e_*); *C_e_* represents the equilibrium concentration of RB221 (mg/L); *q_e_* represents the amount of RB221 adsorbed per gram of adsorbent at equilibrium (mg/g); Δ*S*^0^ (J·mol^−1^·K^−1^) represents the entropy; Δ*H*^0^ (kJ/mol) represents the enthalpy; and *R* represents the gas constant (8.314 J·mol^−1^·K^−1^)

**Figure 7:**
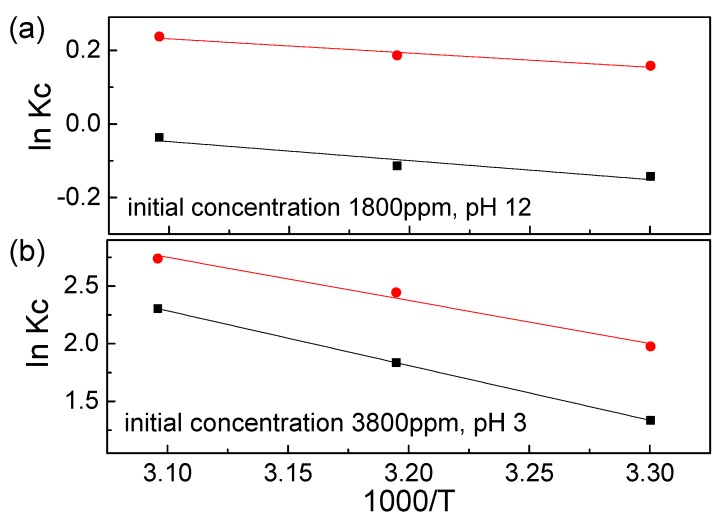
Relationship plots of ln*K_c_* vs. 1/T for determining the thermodynamic parameters under different test conditions: (**a**) at a pH of 12 and an initial RB221 concentration of 1800 mg/L and (**b**) at a pH of 3 and an initial RB221 concentration of 3800 mg/L (■ CS, ● CSGO).

The relationship between ln*K_c_* and 1000/*T* was plotted using the measured data, as shown in Figure 7. According to the equations, the values of the enthalpy Δ*H*^0^ and entropy Δ*S*^0^ were given by the slope and y-intercept, respectively. According to the results in Table 2, the following conclusions are drawn.

1. For adsorption systems with different pH values, both Δ*G*^0^ and Δ*H*^0^ were negative for the adsorption of RB221 by different adsorbents as the temperature increased from 303 to 323 K. The negative values of the two parameters indicate that the adsorption processes of RB221 by the two adsorbent materials were both spontaneous, and there was no need to supply energy externally.

2. A negative Δ*H*^0^ indicates that the adsorption process is exothermic. Alkan et al. [47] reported that the enthalpy change caused by chemisorption is in the range of 40–120 kJ/mol, which is larger than that caused by physisorption. Therefore, because the adsorption heat values—obtained from the tests of adsorption systems with different pH values—were lower than the chemisorption-induced enthalpy changes, it can be inferred that the adsorption observed in this study was due to the effect of physisorption.

3. When the temperature increased from 303 to 323 K at a pH of 3, the maximum adsorption capacities of CS and CSGO decreased from 792.47 and 462.67 mg/L to 344.89 and 230.77 mg/L, respectively.When the temperature increased from 303 to 323 K at a pH of 12, the maximum adsorption capacities of CS and CSGO decreased from 964.196 and 828.886 mg/L to 916.75 and 793.71 mg/L, respectively.

This phenomenon could have resulted from the fact that the adsorption behaviors of the adsorbent materials for RB221 were exothermic adsorption processes, which were mainly caused by the Coulomb electrostatic force between the sulfonate groups on the RB221 molecule and the protonated amine groups on chitosan. Additionally, the H bonding force between the adsorbents and the dye molecules could have contributed to this phenomenon.

### 3.5. Adsorption Kinetics

**Figure 8:**
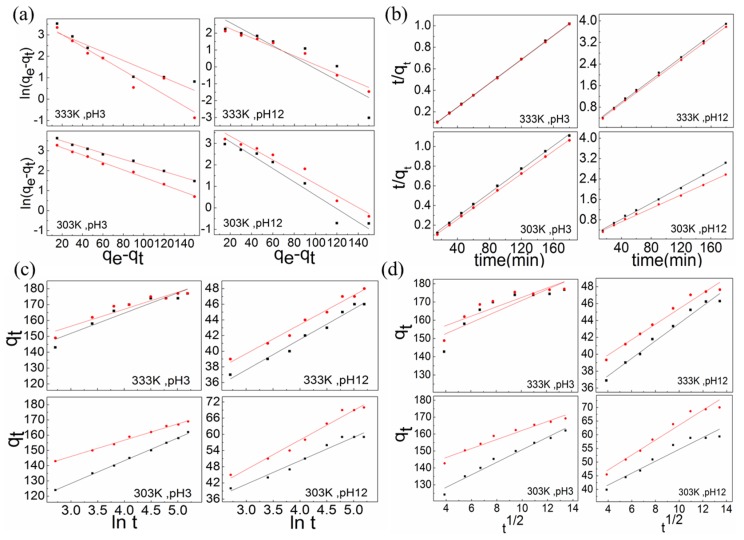
Adsorption kinetics models of the two adsorbents at different temperatures: (**a**) pseudo-first-order model; (**b**) pseudo-second-order model; (**c**) Elovich model; (**d**) Weber–Morris model (■ CS, ● CSGO).

The two most commonly used models in the study of adsorption kinetics are the pseudo-first-order rate and the pseudo-second-order rate model; additionally, the Elovich model and Weber–Morris model are used [41]. The corresponding equations are Equations (14)–(18). In this section, the adsorption kinetics of different adsorbent materials are analyzed and discussed. The adsorption kinetics models for different temperatures are shown in Figure 8.

#### 3.5.1. Pseudo-First-Order Rate Model

(14) $$\log\left(q_{e}-q_{t}\right)=\log q_{e}-\frac{k_{t}}{2.303}t$$

Here, *q_e_* represents the amount of RB221 adsorbed per gram of adsorbent at equilibrium (mg/g), *q_t_* represents the amount of RB221 adsorbed by the adsorbent at time *t* (mg/g), *k*_1_ is the pseudo-first-order rate constant (1/min), and *t* represents time (min).

**Figure 9:**
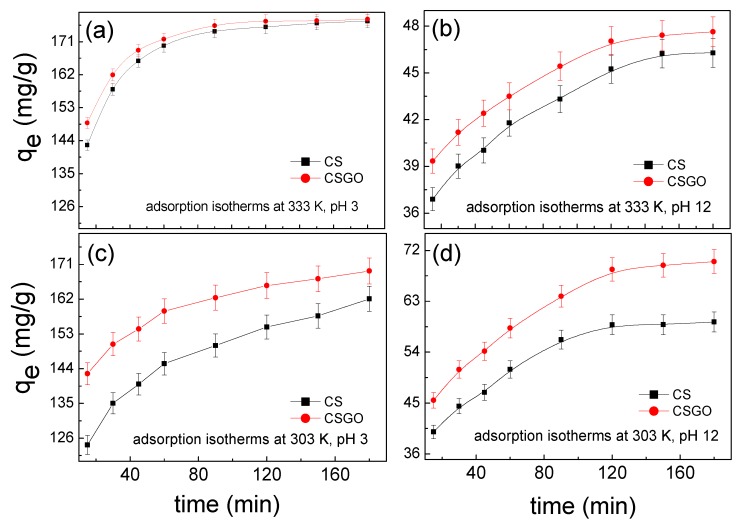
Time variation trends of the dye adsorption capacities of the two adsorbents at different pH values and temperatures: (**a**) pH 3 and 333 K, (**b**) pH 12 and 333 K, (**c**) pH 3 and 303 K, and (**d**) pH 12 and 303 K (■ CS, ● CSGO).

According to the pseudo-first-order rate model, the adsorption kinetics models and behaviors of the two adsorbents can be understood from Table 3 and Figure 9. The pseudo-first-order rate constants (*k*_1_) for the adsorption of RB221 by adsorbents at a pH of 3 and different temperatures were calculated. When the temperature increased from 303 to 333 K, the *k*_1_ values of CS and CSGO changed from 0.9848 and 0.9816 min^−1^ to 0.9802 and 0.9726 min^−1^, respectively. Additionally, the pseudo-first-order rate constants (*k*_1_) for the adsorption of RB221 by adsorbents at a pH of 12 and different temperatures were calculated. When the temperature increased from 303 to 333 K, the *k*_1_ values of CS and CSGO changed from 0.9693 and 0.9727 min^−1^ to 0.9665 and 0.9737 min^−1^, respectively. These results indicate the following. (1) The increase in temperature hindered the adsorption of the dye molecules by the adsorbents. (2) A comparison of the overall adsorption behaviors under the changing acidic and alkaline conditions revealed that the monolayer adsorption capacity of CSGO was slightly higher than that of CS and that CSGO still exhibited a higher adsorption efficiency than CS after the acidic–alkaline condition change.

#### 3.5.2. Pseudo-Second-Order Rate Model

(15) $$\frac{t}{q_{t}}=\frac{1}{k_{2}q_{e}{}^{2}}+\frac{t}{q_{e}}$$

(16) $$h=k_{2}q_{e}{}^{2}$$

Here, *q_e_* represents the amount of RB221 adsorbed per gram of adsorbent at equilibrium (mg/g), *q_t_* represents the amount of Reactive Blue 221 adsorbed by the adsorbent at time *t* (mg/g), *k*_2_ represents the pseudo-second-order rate constant (g/mg·min), and *h* represents the initial adsorption rate of the adsorbent (mg/g·min).

According to the pseudo-second-order rate model, the adsorption kinetics models and behaviors of the two adsorbents can be understood from Table 3 and Figure 9. The initial adsorption rates of adsorbents *h* for RB221 at a pH of 3 and different temperatures were calculated. When the temperature increased from 303 to 333 K, the *h* values of CS and CSGO changed from 166.6667 and 172.4138 mg/g·min to 178.5714 and 181.8182 mg/g·min, respectively. Additionally, the initial adsorption rates of adsorbents *h* for RB221 at a pH of 12 and different temperatures were calculated. When the temperature increased from 303 to 333 K, the *h* values of CS and CSGO changed from 63.6943 and 75.7576 mg/g·min to 48.0769 and 49.2611 mg/g·min, respectively. The initial adsorption rates of the two adsorbents were similar.

The pseudo-second-order rate constants (*k*_2_) for the adsorption of RB221 by the adsorbents at a pH of 3 and different temperatures were calculated. When the temperature increased from 303 to 333 K, the *k*_2_ values of CS and CSGO changed from 0.3573 and 0.1248 g/mg·min to 0.0818 and 0.0583 g/mg·min, respectively. Additionally, the pseudo-second-order rate constants (*k*_2_) for the adsorption of RB221 by the adsorbents at a pH of 12 and different temperatures were calculated. When the temperature increased from 303 to 333 K, the *k*_2_ values of CS and CSGO decreased from 2.5072 and 2.8896 g/mg·min to 1.1715 and 0.7465 g/mg·min, respectively. The foregoing results indicate that the temperature increase was not favorable for the initial adsorption performance of the two adsorbents. Additionally, a comparison of the *R*^2^ values of the linear regressions indicated that this model was more appropriate for analyzing the adsorption kinetics of the two adsorbents.

#### 3.5.3. Elovich Model

(17) $$q_{t}=\frac{1}{\beta }\ln\left(\alpha \beta \right)+\frac{1}{\beta }\ln\left(t\right)$$

Here, *q_t_* represents the amount of RB221 adsorbed by the adsorbent at time *t* (mg/g), *α* represents the initial adsorption rate (mg/g·min), β represents the desorption constant (g/mg), and *t* represents time (min).

According to the Elovich model, the adsorption kinetics models and behaviors of the two adsorbents can be understood from Table 3 and Figure 9. The initial adsorption rates *α* of RB221 by the adsorbents at a pH of 3 and different temperatures were calculated. When the temperature increased from 303 to 333 K, the *α* values of CS and CSGO changed from 4.44 × 10^3^ and 4.34 × 10^4^ mg/g·min to 9.14 × 10^4^ and 1.03 × 10^6^ mg/g·min, respectively. Additionally, the initial adsorption rates (α) of RB221 by the adsorbents at a pH of 3 and different temperatures were calculated. When the temperature increased from 303 to 333 K, the α values of CS and CSGO changed from 52 and 43 mg/g·min to 1.985 × 10^3^ and 1.103 × 10^4^ mg/g·min, respectively. Comparing the initial adsorption rates of the two adsorbents under the changing acidic and alkaline conditions revealed that CSGO exhibited better adsorption performance than CS.

Additionally, the desorption constant (β) values of the two adsorbents for RB221 adsorption at a pH of 3 and different temperatures were calculated. When the temperature increased from 303 to 333 K, the β values of CS and CSGO changed from 0.0677 and 0.0932 to 0.0783 and 0.0925, respectively. At a pH of 12, when the temperature increased from 303 to 333 K, the β values of CS and CSGO changed from 0.1152 and 0.0925 to 0.2450 and 0.2759, respectively. According to these results, during the adsorption process, the two adsorbents desorbed large amounts of dyes as the temperature increased. It is thus inferred that in addition to the Coulomb electrostatic force, the H bonding contributed to the adsorption mechanism, resulting in the observed phenomenon as the temperature increased.

#### 3.5.4. Weber–Morris Model

(18) $$q_{t}=k_{dif}t^{0.5}+C$$

Here, *q_t_* represents the amount of RB221 adsorbed by the adsorbent at time *t* (mg/g), *t* represents time (min), *k_dif_* is the intraparticle diffusion rate constant (mg/g·min), and *C* is a constant indicating the impact of the diffusion of the adsorbent/liquid phase interface on the adsorption process. A larger *C* indicates a larger impact of the diffusion of the solid/liquid interface on the adsorption [48,49].

According to the Weber–Morris model, the adsorption kinetics models and behaviors of the two adsorbents can be understood from Table 3 and Figure 9. The intraparticle diffusion rate constants (*k_dif_*) for RB221 adsorption by the adsorbents at a pH of 3 and different temperatures were calculated. When the temperature increased from 303 to 333 K, the *k_dif_* values of CS and CSGO decreased from 3.7064 and 2.6706 mg/g·min to 3.0179 and 2.5610 mg/g·min, respectively. At a pH of 12, when the temperature increased from 303 to 333 K, the *k_dif_* values of CS and CSGO decreased from 2.1707 and 2.7270 mg/g·min to 1.0364 and 0.9157 mg/g·min, respectively. These results indicate that the temperature changes affected the intraparticle diffusion rates of the adsorbents in the dye.

The changes in the constant *C* for the adsorption of RB221 by the adsorbents at a pH of 3 and different temperatures were examined. When the temperature increased from 303 to 333 K, the *C* values of CS and CSGO changed from 113.7400 and 135.4800 to 140.5400 and 146.8100, respectively. At a pH of 12, when the temperature increased from 303 to 333 K, the *C* values of CS and CSGO changed from 32.9690 and 36.2720 to 33.2940 and 36.2440, respectively. The results indicated that the adsorption processes of the two adsorbents were affected by the temperature changes. A larger C value indicated a delayed impact of the diffusion of the adsorbent/dye solution interface on the adsorption process. Therefore, the CSGO was more significantly affected by the temperature. This could have been due to the graphene structure introduced into its molecular structure, which resulted in its lower intraparticle diffusion rate between the two absorbents.

## 4. Conclusions

A hybrid adsorbent (CSGO) was successfully synthesized using CS and TFGO, and its adsorption performance for RB221 in different acid and alkaline environments (pH ranging from 2 to 12) was investigated. Moreover, the adsorption and removal capacities of unmodified CS and mix-modified CSGO for RB221 were compared at pH values of 2 and 12, respectively. The maximum dye removal capacities of CS and CSGO were measured to be 45.5 and 56.1 mg/g, respectively, at a pH of 2 and 5.4 and 37.2 mg/g, respectively, at a pH of 12. The results indicated that the TFGO structure was successfully introduced into the CSGO adsorbent and that it produced π–π interactions, enhancing the adsorption capacity of CSGO in strong acids and bases. Additionally, the effects of the pH, temperature, and adsorption duration on the adsorption behaviors of unmodified CS and mix-modified CSGO for RB221 were tested. Adsorption kinetics calculations revealed that when the temperature increased from 303 to 333 K, at a pH of 3, the initial adsorption rates of CS and CSGO for RB221 increased from 4.44 × 10^3^ and 4.34 × 10^4^ mg/g·min to 9.14 × 10^4^ and 1.03 × 10^5^ mg/g·min, respectively. At a pH of 12, the initial adsorption rates of CS and CSGO for RB221 increased from 52 and 43 mg/g·min to 1.985 × 10^3^ and 1.04 × 10^4^ mg/g·min, respectively. Thus, at different pH values, CSGO exhibited higher overall adsorption performance than CS. Therefore, CSGO is an effective adsorbent for organic anionic dyes in effluents within a wide pH range and can purify dye effluents in a relatively short period of time.

## Figures and Tables

**Table 1:** Isotherm models and corresponding parameters for the adsorption of RB221 by different adsorbent materials at different temperatures.

pH Value	pH 3				pH 12			
Adsorbents type	CS		CSGO		CS		CSGO	
Temperature (K)	303	323	303	323	303	323	303	323
Langmuir Isotherm								
*Q*_0_ (mg/g)	9.94	16.61	10.53	25.32	1.27	1.32	1.68	1.89
*B* (L/mg)	503.00	602.00	950.00	395.00	2625.00	3789.50	2976.50	2649.50
*R_L_*	6.6 × 10^−7^	5.5 × 10^−7^	3.5 × 10^−7^	8.4 × 10^−7^	3.8 × 10^−7^	2.6 × 10^−7^	3.36 × 10^−7^	3.77 × 10^−7^
*R*_2_	0.9782	0.9801	0.9821	0.9810	0.8730	0.9277	0.9460	0.9587
Freundlich Isotherm								
1/*n*	0.363	0.642	0.613	0.602	0.892	0.895	0.848	0.827
*N*	2.755	1.558	1.631	1.662	1.121	1.117	1.179	1.209
*k_f_* (mg/g)	259.24	82.47	74.11	132.13	1.99	2.13	3.49	4.37
*R*_2_	0.9731	0.9882	0.9732	0.9835	0.8885	0.9084	0.9278	0.9482
Temkin Isotherm								
*K_t_* (dm^3^/g)	1.286	1.263	1.243	1.298	1.193	1.195	1.202	1.206
*b* (J/mol)	0.580	0.277	0.263	0.320	0.553	0.568	0.537	0.574
*R*_2_	0.9729	0.9690	0.9876	0.9712	0.7943	0.8064	0.8244	0.8572

**Table 2:** Thermodynamic parameters for the adsorption processes of RB221 by different adsorbents (test conditions: a pH of 3 and an initial RB221 concentration of 3800 mg/L; a pH of 12 and an initial RB221 concentration of 1800 mg/L).

Adsorbent Type	pH Value	Initial Concentration, *C_0_* (mg/L)	Final Concentration, *C_e_* (mg/L)	T (K)	Final Dye Removal, *C_Ae_* (mg/L)	*Kc*	Thermodynamic Parameter		
							Δ*G*^0^ (KJ/mol)	Δ*H*^0^ (KJ/mol)	Δ*S*^0^ (J/mol·K)
CS	3	3800	792.475	303	3007.525	3.795	−3.360	−39.491	141.421
			522.732	313	3277.268	6.269	−4.777		
			344.89	323	3455.101	10.018	−6.188		
CSGO			462.676	303	3337.324	7.213	−4.978	−31.099	119.264
			303.329	313	3496.671	11.528	−6.362		
			230.772	323	3569.228	15.466	−7.354		
CS	12	1800	964.196	303	835.804	0.867	0.360	−4.277	12.858
			951.055	313	848.945	0.893	0.296		
			916.759	323	883.241	0.963	0.100		
CSGO			828.886	303	971.114	1.172	−0.399	−3.200	11.840
			816.744	313	983.256	1.204	−0.483		
			793.715	323	1006.285	1.268	−0.637		

**Table 3:** Different adsorption kinetics models for the adsorption of RB221 by the different adsorbents.

pH Value	pH 3				pH 12			
Adsorbents Type	CS		CSGO		CS		CSGO	
Temperature (K)	303	333	303	333	303	333	303	333
Pseudo First Order Model								
*k*_1_ (min^−1^)	0.9848	0.9802	0.9816	0.9726	0.9693	0.9665	0.9727	0.9737
*R* ^2^	0.9954	0.8826	0.9968	0.9215	0.9475	0.8361	0.9650	0.9645
Pseudo Second Order Model								
*h* (mg/g·min)	166.6667	178.5714	172.4138	181.8182	63.6943	48.0769	75.7576	49.2611
*k*_2_ (g/mg·min)	0.3573	0.0818	0.1248	0.0583	2.5072	1.1715	2.8896	0.7465
*R* ^2^	0.9990	0.9999	0.9998	0.9999	0.9984	0.9991	0.9978	0.9995
Elovich Model								
*A* (mg/g·min)	4.44 × 10^3^	9.14 × 10^4^	4.34 × 10^4^	1.03 × 10^6^	52	1.985 × 10^3^	43	1.104 × 10^4^
β (g/mg)	0.0677	0.0783	0.0932	0.0925	0.1152	0.2450	0.0925	0.2759
*R* ^2^	0.9973	0.9053	0.9969	0.9114	0.9714	0.9817	0.9807	0.9839
Weber-Morris Model								
*k_dif_* (mg/g·min)	3.7064	3.0179	2.6706	2.5610	2.1707	1.0364	2.7270	0.9157
*C*	113.7400	140.5400	135.4800	146.8100	32.9690	33.2940	36.2720	36.2440
*R* ^2^	0.9723	0.7822	0.9556	0.7923	0.9402	0.9800	0.9668	0.9727

## Supplementary Materials

The following are available online at https://www.mdpi.com/2079-4991/10/4/748/s1, Figure S1: FTIR spectra of GO and TFGO, Figure S2: Raman spectra characteristic analysis of GO and TFGO, Figure S3: TEM images showing the microstructures and dispersion characteristics of GO and TFGO, Figure S4: Adsorption capacities of the CSGO adsorbents with different weight ratios for RB221 with respect to the pH, Figure S5: Photographs of the RB221 solution and CSGO/RB221 powder after RB221 was adsorbed by the CSGO adsorbent. (a) FE-SEM image showing the microstructure of CSGO powder after adsorbing RB221; (b) microstructure of pure RB221 powder, Figure S6: FE-SEM micrographs of TFGO in CS: (a) pristine CS, and (b) CSGO, Table S1: Results of FTIR spectroscopy and the characterization of the functional groups on GO before and after modification (GO vs. TFGO), Table S2: Elemental compositions of GO and TFGO, Table S3: Elemental compositions in CS and CSGO, Table S4: Results of IR spectroscopy and the characterization of the functional groups on CSGO before and after the adsorption of RB2221 (CSGO vs. CSGO/dye), Video S1: Demonstration of the C.I. Reactive Blue 221 (RB221) Dye adsorption of β-chitosan/polyamine functionalized graphene oxide (CSGO) for RB221 dye before and after adsorption.
