# Supplementary material for: Adsorption Performance for Reactive Blue 221 Dye of β-Chitosan/Polyamine Functionalized Graphene Oxide Hybrid Adsorbent with High Acid–Alkali Resistance Stability in Different Acid–Alkaline Environments
Source: Nanomaterials (Basel). 2020 Apr 14;10(4):748. doi: 10.3390/nano10040748 (PMC7221750; doi:10.3390/nano10040748)
Supplement: Supplementary file 1 [file nanomaterials-10-00748-s001.zip › nanomaterials-767620-SI/nanomaterials-767620-SI.pdf]

nanomaterials

Supporting Information

Adsorption Performance for Reactive Blue 221 Dye of β-Chitosan/Polyamine Functionalized Graphene Oxide Hybrid Adsorbent with High Acid–Alkali Resistance Stability in Different Acid–Alkaline Environments

Chih-Wei Chiu *, Ming-Tsung Wu, Chih-Lung Lin, Jia-Wun Li, Chen-Yang Huang, Yu-Chian Soong, Jimmy Chi-Min Lee, William Anderson Lee Sanchez and Hsuan-Yu Lin

Department of Materials Science and Engineering, National Taiwan University of Science and Technology, Taipei 10607, Taiwan

* Corresponding author: Tel: +886-2-2737-6521; Fax: +886-2-2737-6544; E-mail: cwchiu@mail.ntust.edu.tw (C.-W. Chiu)

Structural analysis of GO and TFGO surface graft modification

1. FTIR spectroscopy analysis

The surface of hexagonal benzene ring-shaped GO has high chemical stability, and the interaction force between GO and other media is very weak. Additionally, the van der Waals force between GO sheets is very strong, making GO neither hydrophilic nor oleophilic. Thus, GO agglomerates easily and is hardly compatible with other media and polymers. To overcome these inherent shortcomings of GO and enhance its application potential, its surface must be modified to improve its processability and compatibility. Functional groups can be introduced through surface modification. TFGO is synthesized mainly by grafting polyamine functional groups onto the surface of GO to make its surface possess several amine functional groups. The FTIR spectra of GO and TFGO products, as well as the characterization results for functional groups before and after surface modification, are shown in **Figure S1** and **Table S1**, respectively. The signal strengths of the modified TFGO increased at wavenumbers ranging between 1200 and 1580 cm^{-1}, indicating that polyamines were successfully grafted onto the epoxy group of GO. Additionally, the signal strength at a wavenumber of 3400 cm^{-1} decreased, indicating that polyamines were successfully grafted onto the oxidizing group of GO.

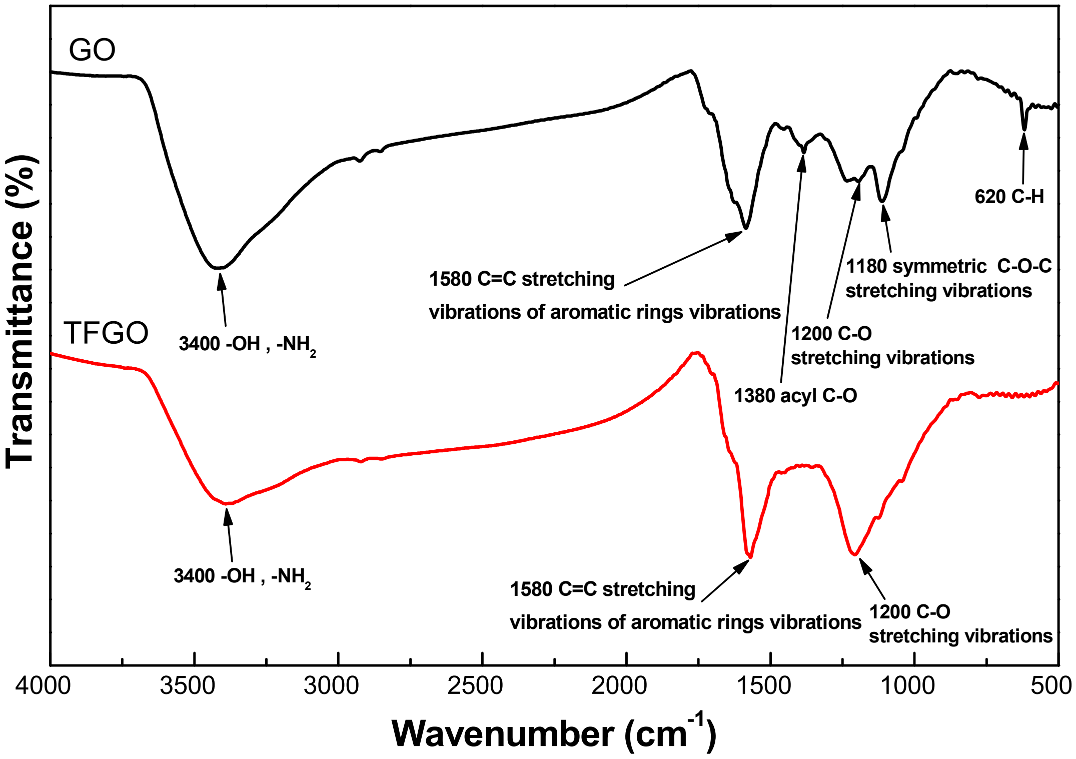

Figure S1. FTIR spectra of GO and TFGO.

Table 1. Results of FTIR spectroscopy and the characterization of the functional groups on GO before and after modification (GO vs. TFGO).

Frequency peaks (cm^{-1})		Functional groups
GO	TFGO	
620	--	C–H
1180	--	symmetric C-O-C stretching vibrations
1200	1200	C-O stretching vibrations
1380	--	acyl C-O
1580	1580	C=C stretching vibrations of aromatic rings vibrations
3400	3400	-OH , $-NH_2$

2. Raman spectroscopy analysis

The wavenumber range of the Raman spectra of GO is 1100–3000 cm^{-1}. Three characteristic peaks corresponding to the sp^2 C material are generated: the D band, G band, and 2D band, at 1350, 1580, and 2680 cm^{-1}, respectively. Among them, the D band is generated by the energy vibration mode of the sp^2 C atom; it is related to the presence of structural defects and edge effects in the hexagonal sp^2 C lattice of graphene, which is referred to as a defect-induced disordered C structure. The G band corresponds to all the stretching vibrations of sp^2 C atom pairs in the rings and chains. This is referred to as a graphite structure with the in-plane vibration of sp^2 C atoms. The 2D band is an overtone related to the limited crystallite size of sp^2 C and represents a second-order Raman scattering process; its shape, width, and location are related to the number of graphene layers. The relative signal intensity of the D band to the G band (I_D/I_G ratio) provides an estimate for the degree of the "disordered carbon structure" in the covalently modified graphene. **Figure S2** shows that the I_D/I_G ratio of GO increased from the original value of 0.99 to 1.14 for TFGO after polyamine modification; the difference was 0.15. This indicates that the epoxy group on the O-containing functional group of GO reacted with TETA, breaking the sp^2 graphene structure and generating sp^3 C atoms, which increased the D band and G band signal intensities. Thus, TETA was successfully introduced into the epoxy functional group of GO.

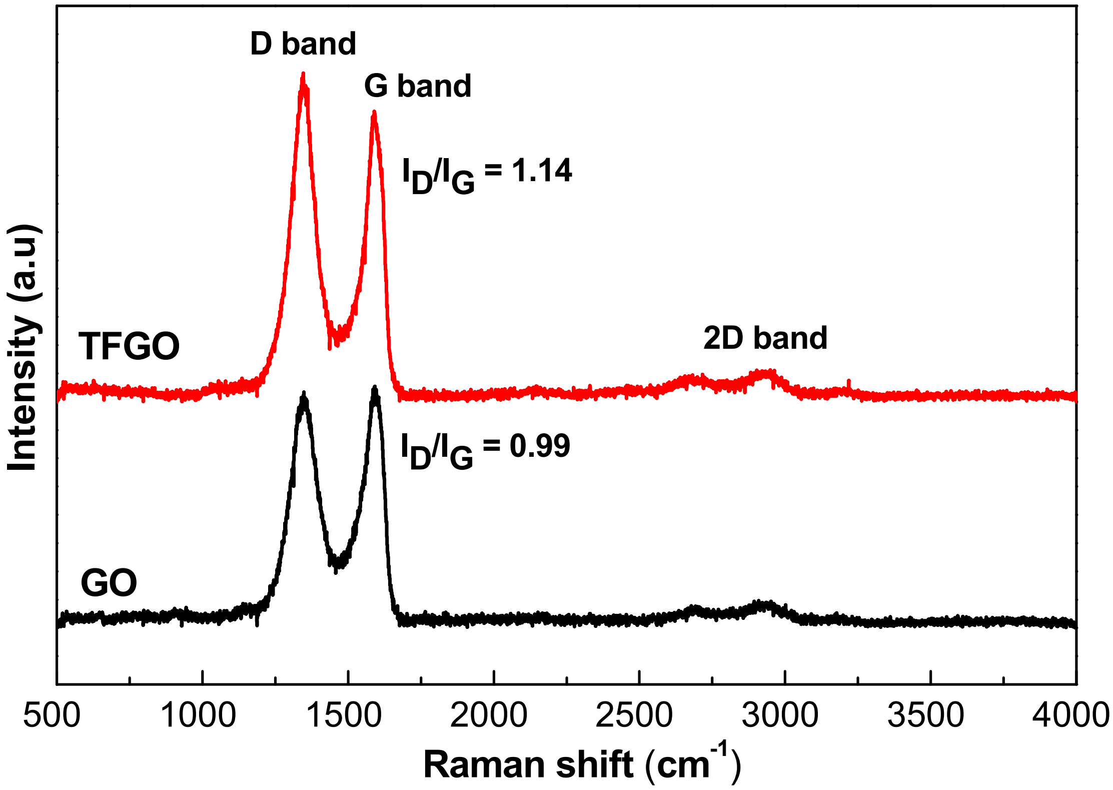

Figure S2. Raman spectra characteristic analysis of GO and TFGO.

3. Elemental analysis

An elemental analyzer was used to analyze the changes in the elemental composition after the GO modification. As indicated by **Table S2**, the N/C ratio of GO was increased from 0% to 12.51% for TFGO after modification. The results indicate that TETA was successfully introduced into the epoxy group on the O-containing functional group on the GO surface.

As shown in **Table S3**, after CS was modified via mixing with TFGO, the O/C ratio increased from the original value of 97.38% to 117.92% for CSGO (increase of 20.54%). This result indicates that the CS was uniformly modified via blending with TFGO.

Table S2. Elemental compositions of GO and TFGO.

Sample	Element (%)					
	C	N	H	O	total	N/C
GO	44.27	0.00	3.06	45.81	93.14	0.00
TFGO	58.75	7.35	4.07	28.28	98.45	12.51

Table S3. Elemental compositions in CS and CSGO.

Sample	Element (%)					
	C	N	H	O	total	O/C
CS	41.72	7.77	8.06	40.63	98.18	97.38
CSGO	39.00	7.37	7.12	45.99	99.48	117.92

4. TEM observations of GO and TFGO microstructures

The differences in the dispersion and microstructures of GO and TFGO are shown in **Figure S3**. The unmodified GO exhibited wrinkled black shadows and layer-to-layer agglomeration. However, the modified TFGO did not have distinct wrinkled black shadows, and the sheet had a relatively transparent and dispersed morphology. Therefore, TETA was successfully introduced into the epoxy functional group of GO.

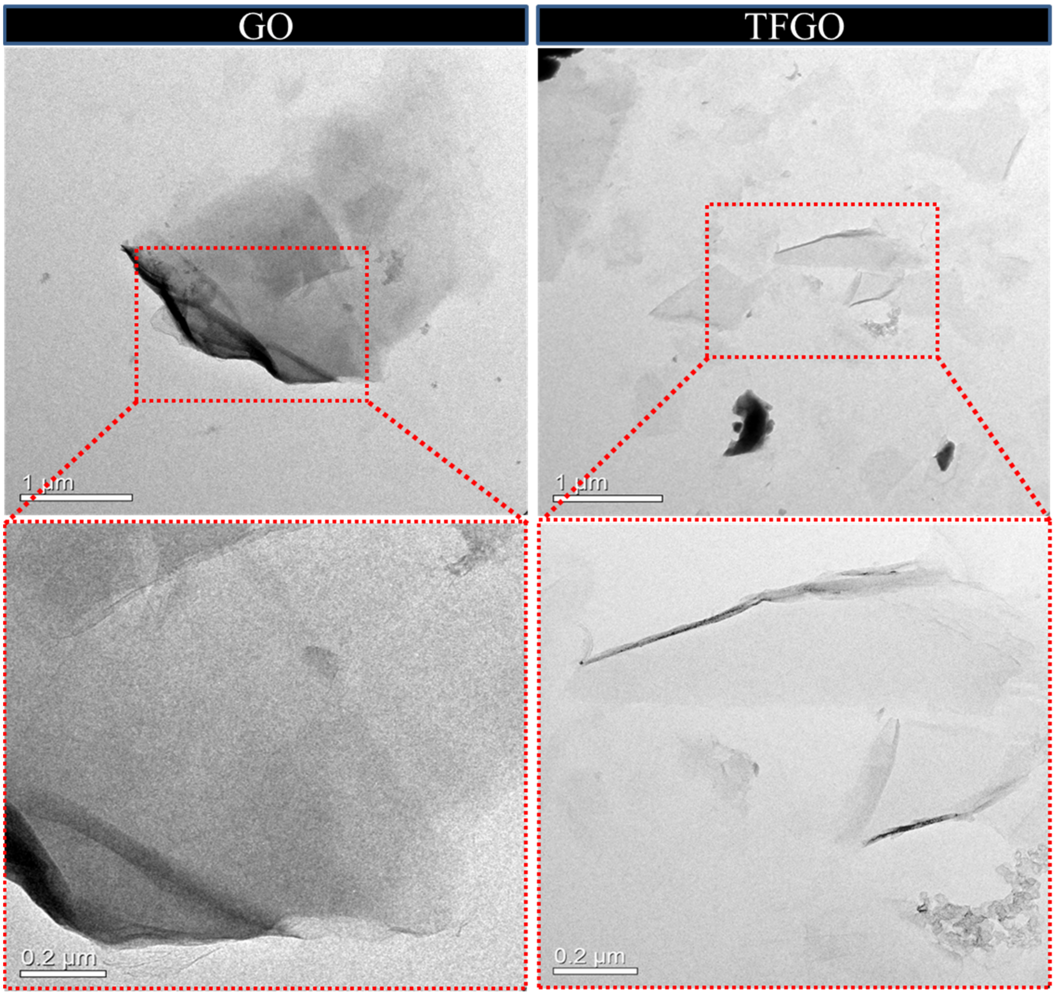

Figure S3. TEM images showing the microstructures and dispersion characteristics of GO and TFGO.

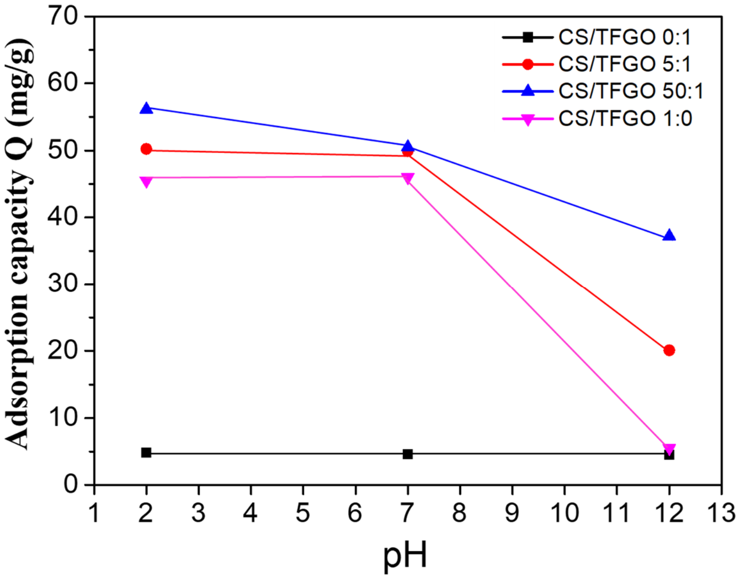

Figure S4. Adsorption capacities of the CSGO adsorbents with different weight ratios for RB221 with respect to the pH.

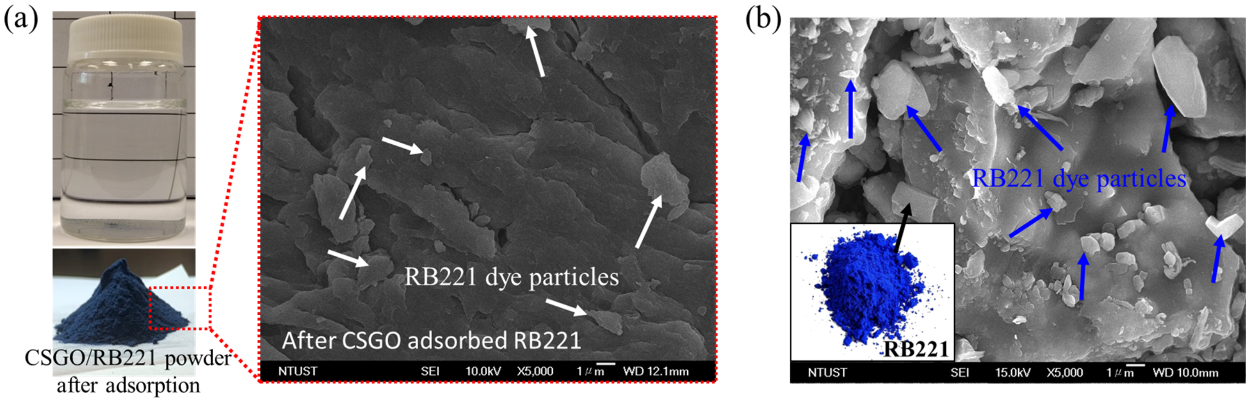

Figure S5. Photographs of the RB221 solution and CSGO/RB221 powder after RB221 was adsorbed by the CSGO adsorbent. (a) FE-SEM image showing the microstructure of CSGO powder after adsorbing RB221; (b) microstructure of pure RB221 powder.

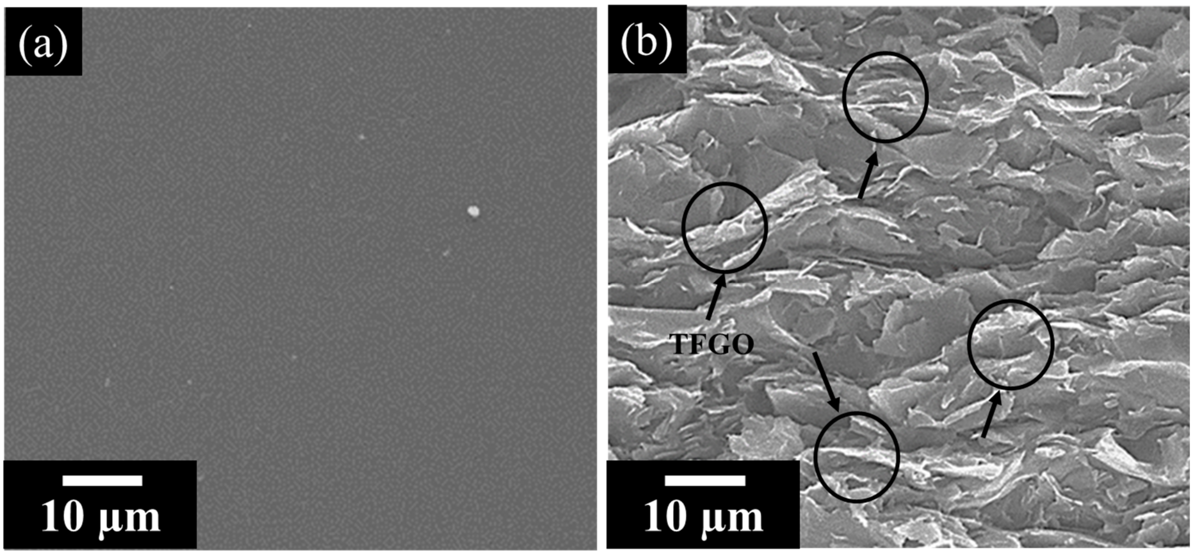

Figure S6. FE-SEM micrographs of TFGO in CS: (a) pristine CS, and (b) CSGO.

Table S4. Results of IR spectroscopy and the characterization of the functional groups on CSGO before and after the adsorption of RB221 (CSGO vs. CSGO/dye).

Frequency peaks (cm^{-1})		Functional groups
CSGO	CSGO/Dye	
660	660	C-H
900	900	C-O
990	990	C–O–C bridge (gluosidic linkage)
1022	1022	primary alcohol.
1080	1080	secondary alcohol
1153	1153	Glycosidic bond
1266	1266	C-N
1322	1322	Amide III band
1380	1380	acyl C-O
1422	1422	C-H
1594	1594	NH_2 band (chitosan)
1642	1642	C=O−NH−CH_3 stretching
2874	2874	C–H stretching
2920	2920	C–H stretching
3400	3400	-OH , -NH_2
